# A randomised controlled trial investigating the effects of Mediterranean diet and aerobic exercise on cognition in cognitively healthy older people living independently within aged care facilities: the Lifestyle Intervention in Independent Living Aged Care (LIILAC) study protocol [ACTRN12614001133628]

**DOI:** 10.1186/s12937-015-0042-z

**Published:** 2015-05-24

**Authors:** Roy J. Hardman, Greg Kennedy, Helen Macpherson, Andrew B. Scholey, Andrew Pipingas

**Affiliations:** 1 Centre for Human Psychopharmacology, Swinburne University of Technology, Melbourne, Australia; 2 Centre for Physical Activity and Nutrition Research, Deakin University, Melbourne, Australia

**Keywords:** Exercise, Diet, Mediterranean, Cognitive, Cognition, Ageing, Aged Care

## Abstract

**Background:**

The rapid ageing of the population is becoming an area of great concern, both globally and in Australia. On a societal level, the cost of supporting an ageing demographic, particularly with their associated medical requirements, is becoming an ever increasing burden that is only predicted to rise in the foreseeable future. The progressive decline in individuals’ cognitive ability as they age, particularly with respect to the ever increasing incidence of Alzheimer’s Disease (AD) and other cognitive complications, is in many respects one of the foundation stones of these concerns. There have been numerous observational studies reporting on the positive effects that aerobic exercise and the Mediterranean diet appear to have on improving cognitive ability. However, the ability of such interventions to improve cognitive ability, or even reduce the rate of cognitive ageing, has not been fully examined by substantial interventional studies within an ageing population.

**Methods:**

The LIILAC trial will investigate the potential for cognitive change in a cohort of cognitively healthy individuals, between the ages of 60 and 90 years, living in independent accommodation within Australian aged care facilities. This four-arm trial will investigate the cognitive changes which may occur as a result of the introduction of aerobic exercise and/or Mediterranean diet into individuals’ lifestyles, as well as the mechanisms by which these changes may be occurring. Participants will be tested at baseline and 6 months on a battery of computer based cognitive assessments, together with cardiovascular and blood biomarker assessments. The cardiovascular measures will assess changes in arterial stiffness and central pulse pressures, while the blood measures will examine changes in metabolic profiles, including brain derived neurotrophic factor (BDNF), inflammatory factors and insulin sensitivity.

**Conclusion:**

It is hypothesised that exercise and Mediterranean diet interventions, both individually and in combination, will result in improvements in cognitive performance compared with controls. Positive findings in this research will have potential implications for the management of aged care, particularly in respect to reducing the rate of cognitive decline and the associated impacts both on the individual and the broader community.

**Trial registration:**

Australia New Zealand Clinical Trial Registry-ACTRN12614001133628

## Background and rationale

The decline in cognitive ability with increasing age has been well established [1–4] and is associated with a reduction of functioning in areas such as attention regulation, processing speed and memory capacity [5]. This phenomenon of cognitive decline is of considerable concern on an individual level. In ageing and elderly populations significant concerns exist regarding the loss of mental faculties and independence of living, which is a cause of considerable psychological distress [1].

On a societal level this phenomenon is also of substantial concern, particularly with the current rapid ageing of the population. The over 60s are the fastest growing age demographic on Earth [6]. It is predicted, that almost one third of the total population of the planet will be aged 60 years or older by 2100 [7], with this percentage predicted to be reached by just 2025 in the Pacific/Oceanic region [8]. In Australia alone, the combined financial cost of formal and informal care of those with cognitive impairment and dementia was estimated to be around AU$8.2 billion in 2010 [9]. This cost is expected to rise to over AU$28 billion by 2040, causing considerable strain on the economy. It has also been predicted that a reduction of just two years in the onset of care requirements due to these age related cognitive issues could result in a 10 % reduction in the overall financial burden [9]. Given the societal and personal consequences, understanding the mechanisms behind the differential rates of cognitive ageing, and designing strategies to prevent or reduce the rate of cognitive decline that are simple and cost effective to implement, are of critical importance.

## What is cognitive ageing?

While there are multiple ways to characterise cognitive ability, it is commonly divided into crystallised intelligence and fluid intelligence. Crystallised intelligence refers to the knowledge and skills that are accumulated over a lifetime [10, 11]. This includes mental functions such as verbal ability, general knowledge and some numerical abilities [2]. Fluid intelligence, often highly associated with cognitive speed [12], refers to the ability to process information, think abstractly and solve problems. This includes aspects of memory, executive functioning and reasoning, and is considered to be largely innately determined [2, 13]. There is general consensus that while crystallised intelligence remains intact until late old age, fluid abilities decline progressively from middle adulthood onwards [14, 15]; and this decline appears to affect a number of fluid cognitive functions simultaneously. However, while the average performance on most fluid cognitive tasks have been found to decline with age, many studies have shown that some older people display comparatively little change, whereas others deteriorate quite rapidly [16, 17] and that this variability increases with increasing age [5].

Earlier research on the relationship between age and cognitive decline indicates that an individual’s cognitive performance increases up until adolescence or early adulthood and then progressively declines. However, the age at which decline begins and the extent of decline varies for different abilities [18]. It is the ageing brain and the consequences of long term effects on the brain that result in speed and memory performance decline [10, 14]. The ageing of the brain may be considered as a progressive and inevitable process potentially related to inflammation, the accumulation of oxidative stress and the related damage to cellular structures of the brain [19]. During normal ageing the brain may suffer morphological and functional deterioration that will potential affect neurotransmission and the alteration of motor and sensory systems, sleep, memory and potential learning [19–22].

A comprehensive review of earlier studies concluded that a slower rate of cognitive ageing could be predicted to a certain degree (along with other factors such as more years of education and an absence of the APOE ε4 allele) by an active lifestyle and better cardiovascular health [10]. This apparent effect of cardiovascular health on the rate of cognitive decline associated with increasing age has also been found by numerous recent studies [23–25]. More specifically, along with avoidance of smoking and limited alcohol consumption, optimisation of cardiovascular health can be most readily achieved through regular exercise and an anti-inflammatory diet focused on fruits and vegetables, such as the Mediterranean diet [26].

## Cognition and exercise

Numerous studies have identified benefits of exercise training for the cognitive functioning of older adults [27]. A meta-analysis, published in 2012, of 29 studies in older adults free from dementia, demonstrated that aerobic exercise interventions, ranging from 8 to 72 weeks, improved cognitive faculties including attention, processing speed, executive function, and memory [28] In a recent Australian study, older participants engaged in moderate aerobic exercise for 50 min three times per week for 6 months [29]. At the end of the study period, individuals in the exercise condition showed enhanced cognition in comparison to those not engaged in the exercise regime.

The cognitive benefits of exercise appear to increase with frequency of exercise [30], and are greatest for moderate (31–45 min) duration exercise sessions [27]. Whilst it has been suggested that the greatest cognitive improvements may occur when aerobic exercise is combined with strength training [28], there is evidence that walking programs may also be effective to improve cognitive function [31]. In a recent study conducted in seniors, half of the participants took part in a 90 min weekly walking program and half served as a control group [32]. After 3 months there was a significant improvement in word fluency, social interaction and motor function in those who participated in the walking program.

Several mechanisms of action have been proposed to account for the cognitive benefits of exercise in older adults. Long term effects of exercise may have indirect effects upon neurocognitive functioning by reducing diabetes, hypertension and cardiovascular disease [33]. Exercise may also have direct effects on brain physiology. There is increasing evidence that extended periods of exercise leads to increased volume of the hippocampus, a brain region vital for memory function, as well as reducing the rate of cerebral grey matter shrinkage [34]. Shorter term mechanisms have also been proposed, due to the identification of cognitive improvements following exercise interventions of several months [28]. For example, exercise may protect against inflammatory processes and regulate growth factor signalling in the brain [33]. Furthermore it has been suggested that exercise may interact with BDNF, a neurochemical which modulates brain plasticity, neuritic outgrowth, synaptic function, and can stimulate adult hippocampal neurogenesis [34].

## Cognition and diet

Dietary factors are considered to have a powerful influence on brain function on a daily basis [35]. With respect to the type of diet that may be of long term benefit, it has been found that people who adhere to a Mediterranean-style diet could reduce the potential for progression from healthy cognitive function to mild cognitive impairment (MCI) and subsequently to dementia [36–39]. Additionally, a diet higher in components of the Mediterranean diet has been found to correlate positively with reduced changes in Mini Mental State Examination (MMSE) scores over five or more years in cognitively healthy people over the age of 65 [40–42]. The Mediterranean diet refers to the dietary practices of populations from the Mediterranean regions. This diet consists predominantly of fruits, vegetables, nuts, cereals, legumes, fish and olive oil, with a moderate intake of alcohol and lower consumption of red meat and poultry. Adherence to the Mediterranean diet has also been linked to lower risk of mortality and vascular events [43], as well a reduction in the incidence of MCI [38].

It has also been found that specific nutritional intake has an impact on overall mental health and function [44]. Antioxidant-rich and flavonol containing components of the Mediterranean diet have been correlated with better performance on spatial working memory in younger women, aged 19 to 30 years, in as little as 10 days [45]. This correlation was also found across multiple measures of memory in people aged 55 to 80 years [46].

Similar to exercise interventions, it is likely that vascular processes and inflammatory pathways may also mediate the relationship between the Mediterranean diet and cognition. An eight week Mediterranean diet intervention has been showed to reduce circulatory markers of inflammation [47, 48]. This may be due to the combined effects of a range of components of the Mediterranean diet. A higher intake of fruit and vegetables has been associated with higher blood nutrient levels, lower oxidative stress and better cognitive function [49]. A high consumption of fruit and vegetables, or a diet that is full of antioxidants, carotenoids, vitamins, fibre and magnesium, has been shown to be beneficial in reducing C-reactive protein (CRP) levels, a marker of inflammation closely linked to cognition [50]. In addition, flavonoids found in fruit and vegetables, and omega-3 polyunsaturated fatty acids found in high quantities in fish, have demonstrated anti-inflammatory, cardio-protective and neuro-protective properties [51, 52].

The cardio-protective nature of the Mediterranean diet has been investigated through an intervention study involving 342 participants. This study found that on a 10 point Mediterranean diet adherence scale, even a 1 point increase in adherence to this type of diet resulted in an 18 % reduction in the relative associated risk of a myocardial infarction [53]. It has also been found that the increased consumption of olive oil and whole grains, that are part of a Mediterranean diet, are both independently associated with a decrease in systolic and diastolic blood pressure [54]. Additionally, another major component of the Mediterranean diet, the increased intake of fish, has also been found to reduce both blood pressure and blood lipids [37, 55]. Finally, it been suggested that the Mediterranean diet may benefit insulin sensitivity and subsequently have a positive effect on decreasing cardiovascular disease [56]. This is further supported by reviews of Mediterranean food patterns and prevention of chronic disease, which have found that greater adherence to the Mediterranean style of diet was associated with a reduction in coronary heart disease [53, 57].

To date, very few studies have conducted randomised controlled based assessment of the effect of Mediterranean diet on health or cognitive outcomes. Recently a 3-arm control randomised study was undertaken using a Mediterranean diet pattern and nuts. The results of this study indicated that the Mediterranean diet has a protective effect on cardiovascular health and also cognitive function [58]. However, the mechanisms by which Mediterranean diet may actually achieve the observed improvements in cognitive performance or improved cardiovascular health have not yet been fully investigated. In particular the effect of this diet on the combination of arterial stiffness, central pulse pressures, circulatory biomarkers of inflammation and changes in BDNF. Additionally, while there have been numerous observational studies, to date no clinical trial of the effects of a Mediterranean diet, in elderly populations, using sensitive tests of specific cognitive functions, has been found.

## Cognition and combined exercise and diet

Exercise can potentially overcome the effects of a poor diet [59] and, in animal trials, exercise has been found to improve cognitive performance and increase hippocampal BDNF in rats with diet induced cognitive decline [60]. Conversely, it has been suggested that diet high in fruit and vegetables, combined with exercise, can assist the brain to cope with several types of insults and ultimately benefit brain function over time [59].

Healthy diet and exercise are associated with a reduced risk of cognitive decline and may provide opportunities to reduce the threat of cognitive decline more effectively than that which is currently understood [61]. With the increasing understanding of molecular biology it has been shown that diet and exercise can affect analogous molecular systems and that this can help lead us to understand a mechanism by which lifestyle, such as exercise and diet may positively influence brain plasticity [59]. Additionally, endothelial dysfunction has been associated with reduced cognitive performance [62], and, in a recent study of healthy older people, a combined Mediterranean diet and exercise intervention was found to provide enduring improvement in vascular endothelial function [63].

However, while there is support for the idea that Mediterranean diet and exercise will combine synergistically to reduce the rate of cognitive decline, there are two main aspects which require further investigation. Firstly, while there is an acknowledged need for a clinical trial of the effects of exercise and diet on cognitive function [61, 64], no such trial has been undertaken as yet. Secondly, the mechanisms by which Mediterranean diet and exercise may affect cognition have yet to be fully investigated. The current study aims to address both of these issues.

## Aims and study hypotheses

The primary aim of the current study is to examine the 6-month effects of increased exercise through walking, change to a Mediterranean diet, and a combination of both, on cognitive performance in an elderly population living independently in aged care facilities. It is hypothesised that measures of cognitive performance will be improved in both the exercise and dietary interventions, relative to the controls, and that the combined diet and exercise intervention will show the greatest improvement of all groups.

As a secondary aim, this study will also investigate the associations between cognitive function and blood based biomarkers and cardiovascular function, which have been proposed as mechanisms, or indicators of mechanisms, that may influence the rate of cognitive ageing. By investigating these variables, the current study aims to identify and target modifiable risk factors, as well as identifying objective biological indicators of the efficacy of interventions that are aimed at ameliorating the rate of cognitive ageing.

## Design and methodology

### Design

This is a randomised, controlled, four-arm parallel groups design. However, given the nature of the interventions, this study is not blinded as both investigators and participants will clearly know what the intervention is, as participants in the lifestyle arms will be actively working towards maintaining the lifestyle change. Participants will be randomly allocated to one of the following four groups:

1. Control
2. Exercise intervention
3. Dietary intervention
4. Combined dietary and exercise intervention

The randomisation sequence will be created by permuting into blocks of 4. While allocation of participants to these groups will be randomised, for reasons of practicality, and to avoid cross contamination between the requirements of each group, when cohabiting couples participate, one person will be randomly allocated to a group and the other will be allocated to the same group.

## Participants

In total, 148 participants who live independently in aged care facilities across a minimum of 15 sites, and who are aged between 60 and 90 years, will participate in this study. These residents live in their own unit or similar accommodation, and are generally physically and mentally capable of caring for themselves in all aspects of daily living. The independent aged care resident population was selected due the generally greater homogeneity of the population and living conditions than that found in the broader community. Furthermore, by focusing on independent living in aged care facilities, this research aims to examine the efficacy of programs aimed at minimising cognitive decline and reducing the rate of transfer to low and high care accommodation.

### Eligibility criteria

To be eligible to participate in the study, participants must be able to speak and read English fluently. Those who are not taking vitamins or herbal supplements as part of a regular regime will be asked to discontinue their use for the duration of the trial, while those who regularly take such vitamins or supplements will be asked to continue taking them in the same manner for the length of the trial. Finally, participants will be required to get the approval of their General Practitioner before random allocation and commencement.

### Ineligibility criteria

Participants will be unable to participate in the trial if they have any significant visual impairment that would prevent them from reading or performing computer based tasks, have a significant neurological or psychiatric disorder, or are unable to walk independently and safely. Additionally, study participants will not be eligible if taking illicit drugs or cognitive enhancing medications. Finally, those with suspected cognitive impairment (defined as a score ≤ 24 on the MMSE), or a significant level of symptoms of depression (a score > 9 on the Geriatric Depression Scale (GDS)), will also be excluded.

This study was ethically approved by the Swinburne University Human Research Ethics Committee (project number 2013/057) and all participants will provide written consent from both themselves and their general practitioner before commencement in the trial.

## Sample size

To date no studies using specific measures of cognition more sensitive than the MMSE have investigated the cognitive effects of a Mediterranean dietary intervention in the elderly. However, numerous trials have examined the effects of exercise fitness interventions. In a meta-analysis conducted to investigate the potential for fitness training to improve cognition in sedentary, but otherwise healthy, older adults, fitness training increased cognitive performance 0.5 SD on average, regardless of the type of cognitive task or the training method [27]. Using Cohen’s effect size criteria, this represents a medium effect size [65]. Although there are no relevant trials which have focussed on the Mediterranean diet, from which to determine an effect size, we have previously identified slightly larger effect sizes on SUCCAB memory measures following several months combined micronutrient, mineral and herbal supplementation in older people with risk factors for cognitive decline [66, 67]. We would anticipate the dietary change condition may exert many of the same benefits as micronutrient dietary supplementation, therefore a comparable effect size may be anticipated. Power analysis was conducted using GPower 3.1.3 to determine the sample size required for a four armed intervention (exercise change only, diet change only, exercise and diet change, no change) using an analysis of variance (ANOVA) design. It was indicated that a total sample of 128 participants would be required to detect a moderate effect size (*f* = 0.30), power of 80 % and a significance level of 5 %. Based on previous trials conducted by the Swinburne University Centre for Human Psychopharmacology, an allowance for a 15 % drop out from the trial has been factored in. Therefore, a total sample of 148 participants will be recruited for this trial, with 37 individuals randomised to each arm of the study.

## Interventions

### Mediterranean diet

Those allocated to one of the diet change groups will be required to record their estimate of their percentage adherence to the Mediterranean diet programme each day, with the goal of reaching and maintaining an 85 % adherence rate over the duration of the study. This programme has been produced in association with a specialist nutritional organisation, and includes a structured four week programme with meal ideas and recipes. Additionally, these participants will be supplied with a standard high-quality extra virgin olive oil to use for the duration of the trial.

### Exercise

While there are a number of types of exercise which are potentially cognitively beneficial, walking has been selected for this study. Regular walking, in particular, has been shown to have cognitive benefits in older cohorts [31, 32]. Additionally, walking does not require supervision or training from a health professional and may be suitable for frail elders or those with some functional limitations. For these reasons a walking program is deemed suitable for older participants and will be practical and cost effective to implement.

Those allocated to a group which includes exercise will be required to walk 4 to 5 times a week. They will follow a graduated program with the aim of walking at least 35–40 min as their aerobic exercise. Any participants who already walk this amount regularly will be asked to increase the amount of walking they do. Participants will be provided with pedometers and will be asked to record the number of minutes walked, distance travelled and the number of steps taken for each walking session.

## Procedure

**Fig. 1:**
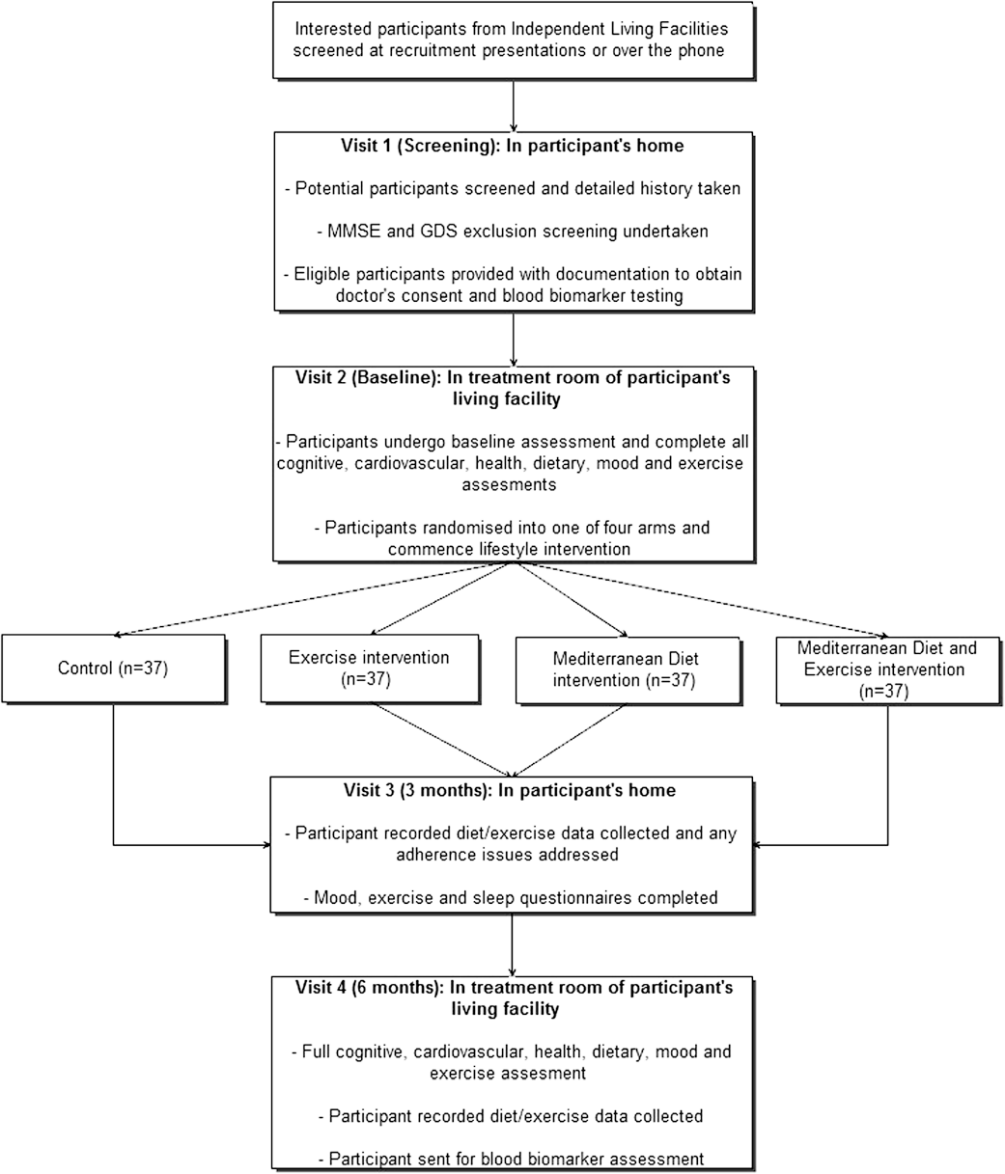
LIILAC protocol flow diagram

Eligible participants will be assessed over four sessions at their living facility. An overview of these sessions is provided in the trial flow diagram (Fig. 1). During the first session, which takes place in the participant’s home, participants are screened for eligibility and assessed with the MMSE and the GDS. Eligible participants are requested to visit their doctor to obtain consent to participate and then to have blood taken for biomarker analysis. Session 2, conducted in a central treatment room in that participant’s living facility, is undertaken once doctor’s consent and blood biomarkers have been obtained. During this visit baseline assessment of all measures are undertaken and participants are informed of the group they have been randomly allocated to. The third session, an in home visit at the 3 month time point, will collect participant recorded diet and/or exercise data where appropriate, and any adherence issues will be discussed. Mood, exercise and sleep questionnaires will also be undertaken at this session. As shown in Fig. 1, the final session will be a full assessment visit, assessing all measures. This will be conducted in a central treatment room of the facility at the 6 month time point. Participant recorded exercise and diet data will also be collected during this session, enabling the investigators to estimate adherence to the intervention.

## Primary outcome

The primary area of interest in this study is the effect of the dietary and exercise interventions on performance in a battery of cognitive measures. The Swinburne University Computerised Cognitive Assessment Battery (SUCCAB) will be undertaken at baseline and at 6 months post baseline. The SUCCAB has been used to assess cognitive performance in previous studies [15, 66, 68]. Reliability and validity assessment has demonstrated that SUCCAB is sensitive to ageing and correlates strongly with memory subtests in the WAIS-IV [15]. The primary outcome will be memory response time calculated as a composite measure from the SUCCAB memory sub-tests.

Additionally, the MMSE will be used to screen for dementia as well as assessing any global effects of the interventions after 6 months.

## Secondary outcomes

Potential mechanisms of action of improvement in cognition through changes in inflammation, glucoregulation, oxidative stress and more direct brain measures such as BDNF, will be examined.

The effects on mood, quality of life, cardiovascular function and overall perceived wellness in these cohorts will also be investigated as potential mechanisms by which exercise and dietary change may have an influence on cognition.

## Measures

Every participant in the study will undertake the same measures, regardless of the intervention they are assigned to.

### Screening

#### Mini Mental State Examination

Participants will be screened for cognitive impairment using the MMSE. Scores 24 or lower may indicate cognitive impairment. Participants scoring 24 or lower will not be eligible for participation.

#### Geriatric Depression Scale

The 30 item version of the GDS [69] will be used to ensure participants are free from depression as late onset depression can be associated with cognitive impairment. Participants scoring above 9 will not be eligible for participation.

### Cognitive assessment

#### Swinburne University Computerised Cognitive Assessment Battery

The SUCCAB is a validated computer based cognitive battery consisting of eight measures that were developed, based on cognitive and neuroimaging literature, to focus on cognitive domains that were most likely to decline with increasing age [15]. This battery uses a simple 5 button interface and has been validated in other studies involving the elderly [70, 71]. The eight measures of cognitive functioning assessed by the SUCCAB consist of Simple and Choice Reaction Times, Immediate and Delayed Recognition, Congruent and Incongruent Stroop colour-words, Spatial Working Memory and Contextual Memory. Computerised measures provide consistency in measurement across participants, and a more automated approach in analysis. The primary outcome will be a composite measure comprised of spatial working memory, immediate, delayed and contextual recognition response time. Secondary outcomes will be response time and accuracy on the individual SUCCAB measures, with the exception of the Congruent Stroop and Reaction Time tasks, as ceiling effects for accuracy are anticipated, response times will be the sole outcome for these tasks.

### Cardiovascular assessment

The effect of the intervention on cardiovascular factors will focus on peripheral and central blood pressures as well as arterial stiffness. These measures will be conducted using applanation tonometry with the SphygmoCor XCEL system. Aortic blood pressure and pulse pressure will be derived automatically, via a brachial blood pressure cuff. Standard brachial blood pressure will also be measured during this process, using an average taken over three recordings. Pulse wave velocity will be derived using a femoral cuff to capture the femoral waveform and tonometer pressure sensor to capture the carotid waveform.

### Biomarker assessment

The blood tests to be undertaken are: BDNF, Cholesterol, Triglycerides, Liver Function Test (LFT), Glucose, Urea, C-Reactive Protein (CRP), Red Cell Folate, Homocysteine, Haemoglobin A1c (HbA1c), Insulin-Like Growth Factor 1 (IGF-1/Somatomedin C) and Vitamins B6, B12 and D. Participants will be asked to fast before these blood tests.

Bloods will be taken by a commercial pathology company or the participant’s medical practitioner. The blood assessment, with the exception of BDNF, is in accordance with a regular medical assessment and represents fairly standard 6 month monitoring conducted by the residents’ medical practitioners.

### Mood and wellness assessment

#### Depression Anxiety Stress Scale (DASS), Profile of Mood States (POMS) and General Health Questionnaire (GHQ)

These self-report mood scales will be used to assess changes in mood across the study. The DASS measures the three related negative emotional states of depression, anxiety and stress [72]. The POMS used to assess transient, fluctuating feelings and enduring affect states allowing participants to report their mood over the past week [73]. The GHQ comprises 60 items and assesses changes in the ability to carry out normal daily functions, somatic symptoms and insomnia, and feelings of anxiety and depression [74].

#### Perceived Wellness Survey

The perceived wellness survey is a set of statements that are designed to provide information about a person’s wellness perceptions [75].

#### Sleep assessment

This study will include the Pittsburgh Sleep Quality Index [76]. This measure assesses ‘components’ of sleep such as disturbances, latency, duration, efficiency, use of sleep medication and daytime dysfunction.

### Dietary assessment

#### Mediterranean diet scale

Adherence to the Mediterranean diet will be assessed by a 10-point Mediterranean-diet scale that incorporated the salient characteristics of this diet. Scores range from 0 to 9, with higher scores indicating greater adherence [77].

#### Food frequency assessment

The Cancer Council Food Frequency Questionnaire [78] will be used to collect additional dietary information. They will also form the basis for nutritional evaluation and to assess adherence to the Mediterranean Diet.

### Exercise assessment

#### The 6 min walk test

The 6 min Walk test will be utilised to indicate baseline fitness as well as improvement in fitness over the study [79]. In this assessment participants will between two markers placed 10 m apart. The objective of this test is to walk for as far as possible in 6 min.

#### Borg assessment

The Modified Borg Scale [80] will be used to rate degree of breathlessness and/or difficult, uncomfortable or laboured breathing as well as fatigue level. Participants will complete the Borg Scale after their 6 min walk test.

#### Community Healthy Activities Model Program for Seniors (CHAMPS) questionnaire

The CHAMPS will be used to the assess baseline physical activity of participants [81]. It will also form part of the assessment of change in overall physical activity across the duration of the trial.

### Demographic and morphometric measures

Age, gender, education and smoking status will be recorded at the commencement of the study. Height, weight, hip and waist circumference will be assessed at baseline and the six month time point, while medication and nutritional supplement information will be collected at all three time points. Additionally, information regarding personal and first degree relative (parents, siblings and children) history of cardiac, stroke or dementia issues will also be recorded.

## Analysis

### Primary outcome

The primary outcome will be response time on the SUCCAB memory measures. Previously benefits to response time on SUCCAB memory measures has been demonstrated following multivitamin [67] and other nutraceutical intervention [68]. Mixed design, repeated measures analysis of co-variance (ANCOVA) will be used to assess treatment-related changes in cognitive performance over the study. Relevant covariates may include group differences in cognitive performance or biomarkers at baseline and co-habitation of participants, where couples are taking part in the intervention trial.

### Secondary outcomes

All other cognitive, biomarker, mood, wellness and exercise measures will be treated as secondary outcomes. Mixed design, repeated measures analysis of covariance (ANCOVA) will be used to assess treatment-related changes in all secondary outcomes over the study. Hierarchical regression will be used to establish the relationship between change in biomarkers and changes in cognitive function. Secondary analysis will examine the pooled effects of the two intervention arms involving the Mediterranean diet versus the control arm and the two intervention arms involving exercise versus the control arm.

## Conclusion

With the rapid ageing of the global population and the commensurate increase in age related cognitive decline, there is an exigent need for broadly available and easily implemented public health interventions that are aimed at ameliorating this issue. There is support for the efficacy of both the Mediterranean style of diet and aerobic exercise interventions in positively affecting cognitive performance in older adults. However, at present, much of the evidence is observational or not balanced by the use of control groups. Additionally, there has been little use of sensitive cognitive measures, covering multiple, specific cognitive domains. Finally, also lacking in the current literature is investigation into the possible mechanisms by which the observed improvements in cognition through diet and exercise, either individually or in combination, may be being achieved. The current trial aims to use clinical trial methodology to test the hypothesis that both Mediterranean diet and walking based aerobic exercise, both independently and in combination, will improve cognitive performance in older people living independently in aged care facilities. Additionally, through blood biomarker, cardiovascular and psychological assessments, this study aims to investigate possible mechanisms by which such improvements may be being achieved. The potential to ameliorate the rate of cognitive decline in older people through achievable lifestyle change may be of substantial importance to public health in an increasingly ageing society.

## Abbreviations

LIILAC: Lifestyle intervention in independent living aged care
ANZCTR: Australia and New Zealand Clinical Trials Registry
AD: Alzheimer’s disease
APOE: Apolipoprotein E
BDNF: Brain derived neurotrophic factor
MCI: Mild cognitive impairment
CRP: C-reactive protein
MMSE: Mini mental state examination
GDS: Geriatric depression scale
SUCCAB: Swinburne University Computerised Cognitive Assessment Battery
LFT: Liver function test
HbA1c: Haemoglobin A1c
IGF-1: Insulin-like growth factor
DASS: Depression anxiety stress scale
POMS: Profile of Mood State
GHQ: General Health Questionnaire
CHAMPS: Community Health Activities Model Program for Seniors

## References

[1] Daffner KR (2010). Promoting successful cognitive aging: a comprehensive review. J Alzheimers Dis.

[2] Deary IJ, Corley J, Gow AJ, Harris SE, Houlihan M, Marioni RE (2009). Age-associated cognitive decline. Br Med Bull.

[3] Levy R (1994). Aging-associated cognitive decline. Int Psychogeriatrics.

[4] Nation DA, Wierenga CE, Delano-Wood L, Jack AJ, Delis DC, Salmon DP (2010). Elevated pulse pressure is associated with age-related decline in language ability. J Int Neuropsychol Soc.

[5] Sachdev P (2003). The aging brain.

[6] United Nations Department of Economic and Social Affairs, Population Division (2007). World Population Ageing 2007.

[7] Lutz W, Sanderson W, Scherbov S (2008). The coming acceleration of global population ageing. Nature.

[8] Scherbov S, Lutz WL, Sanderson WC (2011). The uncertain timing of reaching 8 billion, peak world population, and other demographic milestones. Popul Dev Rev.

[9] Vickland V, Werner J, Morris T, McDonnell G, Draper B, Low L-F (2011). Who pays and who benefits? How different models of shared responsibilities between formal and informal carers influence projections of costs of dementia management. BMC Public Health.

[10] Christensen H (2001). What cognitive changes can be expected with normal ageing?. Aust N Z J Psychiatry.

[11] Horn JL, Cattell RB (1966). Refinement and test of the theory of fluid and crystallized general intelligences. J Educ Psychol.

[12] Christensen H, Kumar R, Sachdev P (2003). Cognitive changes and tha aging brain. The aging brain.

[13] Cattell R, Jenkins J, Patterson D (1961). Fluid and crystallized intelligence. Stud invidual Differ search Intell.

[14] Gunstad J, Paul RH, Brickman AM, Cohen R, Arns M, Roe D (2006). Patterns of cognitive performance in middle-aged and older adults: A cluster analytic examination. J Geriatr Psychiatry Neurol.

[15] Pipingas A, Harris E, Tournier E, King R, Kras M, Stough CK (2010). Assessing the efficacy of nutraceutical interventions on cognitive functioning in the elderly. Curr Top Nutraceutical Res.

[16] Morse CK (1993). Does variability increase with age? An archival study of cognitive measures. Psychol Aging.

[17] Singer T, Verhaeghen P, Ghisletta P, Lindenberger U, Baltes PB (2003). The fate of cognition in very old age: six-year longitudinal findings in the Berlin Aging Study (BASE). Psychol Aging.

[18] Denney NW (1984). A model of cognitive development across the life Span. Dev Rev.

[19] Mariani E, Polidori MC, Cherubini A, Mecocci P (2005). Oxidative stress in brain aging, neurodegenerative and vascular diseases: an overview. J Chromoatogr.

[20] Small G, Silverman D, Siddarth P, Ercoli LM, Miller K, Lavretsky H (2006). Effects of a 14-day healthy longevity lifestyle program on cognition and brain function. Am J Geriatr Psychiatr.

[21] Vaynman S, Ying Z, Wu A, Gomez-Pinilla F (2006). Coupling energy metabolism with a mechanism to support brain-derived neurotropic factor-mediated synaptic plasticity. Neuroscience.

[22] Greenwood PM (2007). Functional plasticity in cognitive aging : review and hypothesis. Neuropsychology.

[23] Hertzog C, Kramer AF, Wilson RS, Lindenberger U (2009). Enrichment effects on adult cognitive development: can the functional capacity of older adults be preserved and enhanced?. Psychol Sci Public Interes.

[24] Scuteri A, Volpe M, Asmar R (2007). Arterial stiffness and cognitive impairment in the elderly. High Blood Press Cardiovasc Prev.

[25] Ram N, Gerstorf D, Lindenberger U, Smith J (2011). Developmental change and intraindividual variability: relating cognitive aging to cognitive plasticity, cardiovascular lability, and emotional diversity. Psychol Aging.

[26] Sierpina VS, Sierpina M, Loera JA, Grumbles L (2005). Complementary and integrative approaches to dementia. South Med J.

[27] Colcombe S, Kramer AF (2003). Fittness effects on the cognitive function of older adults: a meta-analytic study. Psychol Sci.

[28] Smith PJ, Blumenthal JA, Hoffman BM, Strauman TA, Welsh-bohmer K, Jeffrey N (2011). Aerobic exercise and neurocognitive performance: a meta-analytic review of randomized controlled trials. Psychosom Med.

[29] Lautenschlager NT, Cox KL, Flicker L, Foster JK, van Bockxmeer FM, Xiao J (2008). Effect of physical activity on cognitive function in older adults at risk for Alzheimer disease: a randomized trial. JAMA.

[30] Masley S, Roetzheim R, Gualtieri T (2009). Aerobic exercise enhances cognitive flexibility. J Clin Psychol Med Settings.

[31] Rolland Y, van Kan GA, Vellas B (2010). Healthy brain aging : role of exercise and physical activity. Clin Geriatr Med.

[32] Maki Y, Ura C, Yamaguchi T, Murai T, Isahai M, Kaiho A (2012). Effects of intervention using a community-based walking program for prevention of mental decline: a randomized controlled trial. J Am Geriatr Soc.

[33] Cotman CW, Berchtold NC (2007). Physical activity and the maintenance of cognition: learning from animal models. Alzheimers Dement.

[34] Ahlskog JE, Geda YE, Graff-Radford NR, Petersen RC (2011). Physical exercise as a preventive or disease-modifying treatment of dementia and brain aging. Mayo Clin Proc.

[35] Gómez-Pinilla F (2010). Brain foods : the effects of nutrients on brain function. Nat Rev Neurosci.

[36] Middleton LE, Yaffe K (2010). Targets for the prevention of dementia. J Alzheimers Dis.

[37] Scarmeas N, Stern Y, Tang M, Mayeux R, Luchsinger JA (2006). Mediterranean diet and risk for alzheimer’s disease. Ann Neurol.

[38] Scarmeas N, Stern Y, Mayeux R, Manly JJ, Schupf N, Luchsinger JA (2009). Mediterranean diet and mild cognitive impairment. Arch Neurol.

[39] Singh B, Parsaik A, Mielke M, Erwin P, Knopman D, Petersen R (2014). Association of mediterranena diet with mild cognitive impairment and Alzheimer’s disease: a systematic review and meta-analysis. J Alzheimers Dis.

[40] Féart C, Samieri C, Rondeau V, Amieva H, Portet F, Dartigues J-F (2009). Adherence to a Mediterranean diet, cognitive decline, and risk of dementia. JAMA.

[41] Psaltopoulou T, Kyrozis A, Stathopoulos P, Trichopoulos D, Vassilopoulos D, Trichopoulou A (2008). Diet, physical activity and cognitive impairment among elders : the EPIC-Greece cohort (European Prospective Investigation into Cancer and Nutrition). Public Health Nutr.

[42] Tangney CC, Kwasny MJ, Li H, Wilson RS, Evans DA, Morris MC (2011). Adherence to a Mediterranean-type dietary pattern and cognitive decline in a community population. Am J Clin Nutr.

[43] Gardener S, Gu Y, Rainey-Smith SR, Keogh JB, Clifton PM, Mathieson SL (2012). Adherence to a Mediterranean diet and Alzheimer’s disease risk in an Australian population. Transl Psychiatr.

[44] Zainuddin MSA, Thuret S (2012). Nutrition, adult hippocampal neurogenesis and mental health. Br Med Bull.

[45] McMillan L, Owen L, Kras M, Scholey A (2011). Behavioural effects of a 10-day Mediterranean diet. Results from a pilot study evaluating mood and cognitive performance. Appetite.

[46] Valls-Pedret C, Lamuela-Ravent RM, Quintana M, Corella D, Pint X, Angel M (2012). Polyphenol-rich foods in the Mediterranean diet are associated with better cognitive function in elderly subjects at high cardiovascular risk. J Alzheimers Dis.

[47] Frisardi V, Panza F, Seripa D, Imbimbo BP, Vendemiale G, Pilotto A (2010). Nutraceutical properties of Mediterranean diet and cognitive decline: possible underlying mechanisms. J Alzheimers Dis.

[48] Hermsdorff HHM, Zulet MA, Abete I, Martinez JA (2009). Discriminated benefits of a Mediterranean dietary pattern within a hypocaloric diet program on plasma RBP4 concentrations and other inflammatory markers in obese subjects. Endoer.

[49] Polidori MC, Praticó D, Mangialasche F, Mariani E, Aust O, Anlasik T (2009). High fruit and vegetable intake is positively correlated with antioxidant status and cognitive performance in healthy subjects. J Alzheimers Dis.

[50] Ajani UA, Ford ES, Mokdad AH (2004). Dietary fiber and C-reactive protein: findings from national health and nutrition examination survey data. J Nutr.

[51] Ruxton CHS, Reed SC, Simpson MJA, Millington KJ (2004). The health benefits of omega-3 polyunsaturated fatty acids: a review of the evidence. J Hum Nutr Diet.

[52] Young JM, Shand BI, McGregor PM, Scott RS, Frampton CM (2006). Comparative effects of enzogenol and vitamin C supplementation versus vitamin C alone on endothelial function and biochemical markers of oxidative stress and inflammation in chronic smokers. Free Radic Res.

[53] Martinez-Gonzalez MA, Bes-Rastrollo M, Serra-Majem L, Lairon D, Estruch R, Trichopoulou A (2009). Mediterranean food pattern and the primary prevention of chronic disease : recent developments. Nutr Rev.

[54] Psaltopoulou T, Naska A, Orfanos P, Trichopoulos D, Mountokalakis T, Trichopoulou A (2004). Olive oil, the Mediterranean diet, and arterial blood pressure : The Greek European Prospective Investigation into Cancer and Nutrition (EPIC) study. Am J Clin Nutr.

[55] Serra-Majem L, Roman B, Estruch R (2006). Scientific evidence of interventions using the mediterranean diet : a systematic review. Nutr Rev.

[56] Trichopoulou A, Corella D, Martı MA, Soriguer F, Ordovas JM (2006). The Mediterranean diet and cardiovascular epidemiology. Nutr Rev.

[57] Dilis V, Katsoulis M, Lagiou P, Trichopoulos D, Naska A, Trichopoulou A (2012). Mediterranean diet and CHD : the Greek European Prospective Investigation into Cancer and Nutrition cohort. Br J Nutr.

[58] Martínez-Lapiscina EH, Galbete C, Corella D, Toledo E, Buil-Cosiales P, Salas-Salvado J (2014). Genotype patterns at CLU, CR1, PICALM and APOE, cognition and Mediterranean diet: the PREDIMED-NAVARRA trial. Genes Nutr.

[59] Gomez-Pinilla F (2006). The impact of diet and exercise on brain plasticity and disease. Nutr Health.

[60] Noble EE, Mavanji V, Little MR, Billington CJ, Kotz CM, Wang C (2014). Exercise reduces diet-induced cognitive decline and increases hippocampal brain-derived neurotrophic factor in CA3 neurons. Neurobiol Learn Mem.

[61] Nash DT (2007). Nutritional and exercise aspects of cognitive impairment. J Clin Lipidol.

[62] Waldstein SR, Rice SC, Thayer JF, Najjar SS, Scuteri A, Zonderman AB (2008). Pulse pressure and pulse wave velocity are related to cognitive decline in the Baltimore Longitudinal Study of Aging. Hypertension.

[63] Klonizakis M, Alkhatib A, Middleton G (2014). Long-term effects of an exercise and Mediterranean diet intervention in the vascular function of an older, healthy population. Microvasc Res.

[64] Meeusen R (2014). Exercise, nutrition and the brain. Sport Med.

[65] Cohen J (1992). Quantitative methods in psychology. Psychol Bull.

[66] Harris E, Macpherson H, Vitetta L, Kirk J, Sali A, Pipingas A (2012). Effects of a multivitamin, mineral and herbal supplement on cognition and blood biomarkers in older men: a randomised, placebo-controlled trial. Hum Psychopharmacol.

[67] Macpherson H, Ellis KA, Sali A, Pipingas A (2012). Memory improvements in elderly women following 16 weeks treatment with a combined multivitamin, mineral and herbal supplement: a randomized controlled trial. Psychopharmacology (Berl).

[68] Pipingas A, Silberstein RB, Vitetta L, Rooy C, Van VE, Young JM (2008). Improved cognitive performance after dietary supplementation with a Pinus radiata bark extract formulation. Phyther Res.

[69] Parmelee PA, Katz IR (1990). Geriatric depression scale. J Am Geriatr Soc.

[70] Stough CK, Pase MP, Cropley V, Myers S, Nolidin K, King R (2012). A randomized controlled trial investigating the effect of Pycnogenol and Bacopa CDRI08 herbal medicines on cognitive, cardiovascular, and biochemical functioning in cognitively healthy elderly people: the Australian Research Council Longevity Intervention. Nutr J.

[71] Simpson T, Camfield D, Pipingas A, Macpherson H, Stough C (2012). Improved processing speed: online computer-based cognitive training in older adults. Educ Gerontol.

[72] Brown TA, Chorpita BF, Korotitsch W, Barlow DH (1997). Psychometric properties of the depression anxiety stress scale (DASS) in clinical samples. Behav Res Ther.

[73] McNair DM, Lorr M, Droppleman LF (1992). Profile of Mood States (POMS)-revised Manual.

[74] Goldberg DP, Williams PA (2006). User’s Guide to the General Health Questionnaire: GHQ.

[75] Adams T, Bezner J, Steinhardt M (1997). The conceptualization and measurement of perceived wellness: integrating balance across and within dimensions. Am J Heal Promot.

[76] Buysse DJ, Reynolds CF, Monk TH, Berman SR, Kupfer DJ (1989). The Pittsburgh Sleep Quality Index: a new instrument for psychiatric practice and research. Psychiatry Res.

[77] Trichopoulou A, Costacou T, Bamia C, Trichopoulos D (2003). Adherence to a mediterranean diet and survival in a greek population. N Engl J Med.

[78] Giles G, Ireland P (1996). Dietary Questionnaire for Epidemiological Studies (Version 2).

[79] Troosters T, Gosselink R, Decramer M (1999). Six minute walking distance in healthy elderly subjects. Eur Respir J.

[80] Borg GAV (1998). Borg’s perceived exertion and pain scales.

[81] Stewart AL, Mills KM, King AC, Haskell WL, Gillis D, Ritter PL (2001). CHAMPS physical activity questionnaire for older adults: outcomes for interventions. Med Sci Sports Exerc.

